# Cardiac dysfunction during adverse maternal outcomes in hypertensive disorders of pregnancy

**DOI:** 10.1111/aogs.70103

**Published:** 2025-11-17

**Authors:** Veronica Giorgione, Jamie Kitt, Paul Leeson, Asma Khalil, Jamie O'Driscoll, Basky Thilaganathan

**Affiliations:** ^1^ Fetal Medicine Unit St George's University Hospitals NHS Foundation Trust London UK; ^2^ Oxford Cardiovascular Clinical Research Facility, Division of Cardiovascular Medicine, Radcliffe Department of Medicine University of Oxford Oxford UK; ^3^ Vascular Biology Research Centre, Molecular and Clinical Sciences Research Institute St George's University of London London UK; ^4^ Department of Cardiology St George's University Hospitals NHS Foundation Trust London UK; ^5^ Diabetes Research Centre, College of Life Sciences University of Leicester Leicester UK

**Keywords:** adverse maternal outcomes, hypertension, pre‐eclampsia, pregnancy, transthoracic echocardiography

## Abstract

**Introduction:**

Hypertensive disorders of pregnancy are associated with significant cardiac remodeling and diastolic dysfunction during pregnancy and are important contributors to maternal morbidity and mortality. Whether acute adverse maternal outcomes during hypertensive disorders of pregnancy are associated with abnormal left ventricular geometry and function has not been widely studied.

**Material and Methods:**

A prospective observational study was conducted on 255 women with hypertensive disorders of pregnancy who underwent transthoracic echocardiography during the peripartum period. Maternal echocardiographic parameters, including left ventricular morphology and function, were analyzed to determine their association with composite adverse maternal outcomes by univariate and multivariate analyses. The composite adverse maternal outcome was defined as at least one of the following: admission to a high dependency unit (an intermediate‐care ward, providing enhanced cardiac monitoring), acute renal injury, adverse cardiopulmonary events, stroke, and disseminated intravascular coagulation.

**Results:**

Adverse maternal outcomes occurred in 68 (26.7%) participants. Women with adverse outcomes had significantly higher left atrial volume index (28.8 [23.4–32.3] mL/m^2^ vs. 26.6 [22.2–30.9] mL/m^2^, *p* = 0.045) and E/e' ratio (7.8 [6.6–9.2] vs. 7.0 [5.9–8.1], *p* = 0.002) compared to those without complications. Other diastolic indices, namely, mitral inflow E/A and tissue‐Doppler e' velocities at the lateral and septal mitral annulus, showed no statistically significant between‐group. In multivariable analysis, both left atrial volume index and E/e' ratio remained independently associated with adverse maternal outcomes after adjusting for maternal factors and clinical variables. Right ventricular indices, such as tricuspid annular plane systolic excursion and systolic velocity S', were independently associated with adverse maternal outcomes, while fractional area change remained unchanged, indicating hyperkinetic circulatory adaptation rather than enhanced intrinsic right systolic function.

**Conclusions:**

Cardiac abnormalities, particularly in left ventricular diastolic function and in right ventricular function, are more common in women with adverse maternal outcomes in hypertensive disorders of pregnancy than in hypertensive women without adverse maternal outcomes. Further studies are needed to determine whether these echocardiographic abnormalities could help identify women at increased risk of complications.

## Graphical abstract

In pregnancies complicated by hypertensive disorders, peripartum adverse maternal outcomes are associated with diastolic cardiac dysfunction. Elevated E/e' ratio and left atrial volume index highlight the need for targeted cardiovascular monitoring and long‐term follow‐up.

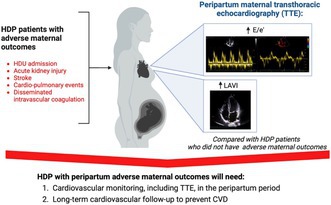

**Abbreviations**
BMI: body mass index
E/e': ratio of early diastolic transmitral flow velocity to early diastolic mitral annular velocity
FAC: fractional area change
HDP: hypertensive disorders of pregnancy
LAVI: left atrial volume index
LVMI: left ventricular mass index
MAP: mean arterial pressure
PCR: protein‐to‐creatinine ratio
TAPSE: tricuspid annular plane systolic excursion
TTE: transthoracic echocardiography

**Key message**

Peripartum adverse maternal outcomes in women with hypertensive disorders of pregnancy are associated with maternal diastolic cardiac dysfunction. Enhanced peripartum cardiovascular monitoring, including maternal transthoracic echocardiography, may be required for these women to optimize care and to mitigate immediate complications during this critical period.

## 1 INTRODUCTION

Hypertensive disorders of pregnancy (HDP), including pre‐eclampsia, gestational hypertension, and chronic hypertension, are significant contributors to maternal and perinatal morbidity and mortality, affecting approximately 5%–10% of pregnancies with an increasing trend globally.^1^, ^2^ In particular, between 5% and 20% of women who develop HDP early in pregnancy can face life‐threatening medical complications, such as cerebrovascular hemorrhage, pulmonary edema, acute kidney injury, liver rupture, and eclampsia.^3^ Worldwide, 10%–15% of direct maternal deaths are associated with HDP, and mortality is higher in patients with heart disease.^4^, ^5^ Although the involvement of the maternal cardiovascular system in HDP patients has been extensively investigated, to date, no clinical guidelines have formally recommended the assessment of maternal hemodynamic or cardiac function during pregnancy.^6^, ^7^ Transthoracic echocardiography (TTE) can provide valuable insights into left ventricular morphology and function in pregnant women. Several echocardiographic studies have highlighted adverse left ventricular remodeling as well as diastolic and systolic dysfunction in pregnancies complicated by hypertension, with a dose–response relationship in echocardiographic findings across the HDP disease spectrum.^8^, ^9^, ^10^ However, few studies have determined whether the occurrence of peri‐partum adverse maternal outcomes during HDP is associated with specific echocardiographic abnormalities that could serve as early markers of risk.^11^, ^12^ Therefore, this study aimed to assess the association between maternal cardiac morphology and function and the development of adverse maternal outcomes in a large cohort of women with HDP, to explore whether echocardiographic parameters may help identify those at increased risk and guide enhanced monitoring strategies.

## 2 MATERIAL AND METHODS

This study is a secondary analysis of a prospective cohort in which women were enrolled during the peripartum period (before delivery or within 1 week postpartum) and followed for 4 months postpartum. It was conducted at St George's University Hospital NHS Foundation Trust between 2019 and 2021. In the present analysis, we included only women who underwent TTE in the peripartum period, and we compared echocardiographic parameters between those who did and those who did not subsequently develop adverse maternal outcomes. The primary results of this study have been previously published.^13^, ^14^, ^15^ These echocardiographic data were obtained only for research purposes, and they did not influence patients' clinical management. Data from before and shortly after delivery were pooled because our previous findings showed no significant changes in cardiac geometry or function when comparing paired TTE data performed before delivery with data within 1 week postdelivery.^15^ However, a sensitivity analysis was performed where only women with TTE before delivery were included.

### 2.1 Study population

The HDP cohort included women with singleton pregnancies complicated by HDP, such as chronic hypertension, pre‐eclampsia, or gestational hypertension, recruited from the Maternity Department. They were enrolled either before delivery or within 1 week postpartum. HDP types were diagnosed according to the criteria established by the International Society for the Study of Hypertension in Pregnancy.^16^ HELLP syndrome was defined when the following criteria were met: liver transaminases >70 IU/L, or twice the upper limit of normal concentration, platelet <100 × 10^9^/L, hemolytic signs like elevated total bilirubin, elevated LDH, and schistocytes on a peripheral blood smear. Hypertension in HDP pregnancies was managed per NICE guidelines using labetalol, nifedipine, or methyldopa based on clinical preferences.^17^ Pregnancies affected by fetal genetic syndromes, fetal abnormalities, or known maternal cardiac conditions were excluded. Data about pregnancy and delivery outcomes were collected from patients' medical records. The last laboratory test results were also collected, including platelet count (10^9/L), creatinine (μmol/L), alanine aminotransferase (ALT, U/L), and urinary protein to creatinine ratio (PCR, mg/mmol) performed before delivery.

### 2.2 Echocardiography measures

Anthropometric measurements, blood pressure (BP), and maternal TTE were performed. Body mass index (BMI, kg/m^2^) was calculated as weight (kg) divided by height squared (m^2^), and body surface area (m^2^) was calculated using the formula: 0.007184*height (cm)^0.725^*weight(kg)^0.425^. Mean arterial pressure (MAP, mmHg) was calculated as (2 * diastolic BP + systolic BP)/3. TTE was performed at rest in the left lateral decubitus position using a GE Vivid E95 with an M5Sc‐D probe (GE Healthcare, Horten, Norway), with analysis conducted using EchoPAC version 203 (GE Healthcare). Measurements adhered to international guidelines.^18^, ^19^, ^20^, ^21^ From the parasternal long‐axis view, the interventricular septum (IVS) diameter, left ventricular end‐diastolic diameter (LVEDd), and left ventricular posterior wall thickness (PWT) were measured. Left ventricular mass (LVM) was calculated using the formula: 0.8 * (1.04 * (LVEDd + PWT + IVS)^3^ − LVEDd^3^) + 0.6. LVM index (LVMI, g/m^2^) was obtained by indexing for body surface area. Relative wall thickness was calculated as 2× PWT/LVEDd. Left atrial volume index (LAVI) was measured at end ventricular systole and indexed for body surface area. Left ventricular end‐diastolic and end‐systolic volumes and ejection fraction (%) were measured using the biplane summation‐of‐disks method. Pulsed wave Doppler assessed mitral valve flow, including peak early diastolic (E‐wave) velocity, atrial contraction (A‐wave) velocity, E/A ratio, and deceleration time. Tissue Doppler imaging measured lateral and septal mitral annulus velocities (e', m/s). The speckle‐tracking analysis provided global longitudinal strain (%) values from the apical 2‐, 3‐, and 4‐chamber views. Right ventricular (RV) systolic function was assessed using: (1) Tricuspid annular plane systolic excursion (TAPSE) measured by M‐mode at the lateral tricuspid annulus in the apical four‐chamber view (mm); (2) tissue‐Doppler systolic velocity S' at the lateral tricuspid annulus (m/s); and (3) RV fractional area change (FAC, %) calculated as [(end‐diastolic area − end‐systolic area)/end‐diastolic area] × 100.

### 2.3 Maternal outcomes

The composite adverse maternal outcome was defined as the occurrence of at least one of the following events: admission to a high dependence unit (HDU) for cardiac monitoring or hourly monitoring; acute kidney injury defined as creatinine>100 μmol/L antenatally or >130 μmol/L postnatally or need for dialysis; adverse cardiac events, such as acute coronary syndrome, pulmonary oedema, or peripartum cardiomyopathy; hemorrhagic or ischemic stroke; and disseminated intravascular coagulation.^22^ The HDU provides enhanced monitoring (continuous cardiac rhythm monitoring with pulse oximetry and automated blood pressure measurements, strict fluid balance) and noninvasive treatments (oxygen therapy, intravenous antihypertensives, magnesium sulfate, diuretics, fluids, and blood products). Per departmental protocol, admission to the obstetric HDU was indicated for persistent severe hypertension requiring IV therapy, magnesium sulfate administration, respiratory compromise or pulmonary oedema, acute kidney injury/oliguria, neurological symptoms, hemodynamic instability, or a requirement for continuous ECG/SpO₂ monitoring with hourly blood pressure and urine output measurements. The composite adverse maternal outcome was coded as “yes” if any component occurred; women with multiple components were counted once in the composite.

### 2.4 Statistical analysis

Continuous variables were expressed as medians with interquartile ranges (IQR), and categorical variables as *n* (%).

In the descriptive analysis, between‐group imbalance of maternal and laboratory variables was summarized with standardized mean differences (SMD) based on log‐transformed means for skewed continuous variables and on group proportions for categorical variables.

In the univariate analysis, echocardiographic continuous variables were analyzed with the Mann–Whitney U‐test. Logistic regression models assessed the association between cardiac parameters and composite maternal adverse outcomes. Multivariate analysis was adjusted for maternal factors (maternal age, ethnicity, BMI, MAP, and pre‐existing comorbidities) and the timing of TTE. Relative risks (RRs) were also calculated by using modified Poisson (log‐link) regression with robust (sandwich) standard errors and presented in the supplementary Table 1. Ordinal logistic regression was used to explore the association of TTE measurements and severity of maternal adverse outcomes that were classified as absent, one and two or more adverse maternal outcomes. Statistical significance was set at *p* < 0.05. Analyses were conducted using SPSS software (version 27.0, SPSS Inc., Chicago, IL, USA).

## 3 RESULTS

The study cohort included 255 pregnancies complicated by HDP (Table 1). The median (IQR) maternal age was 33.8 (29.9–37.3) years, and the first‐trimester median (IQR) BMI was 27.0 (23.1–31.2) kg/m^2^. Women identified themselves as non‐white ethnicity in 39.6% (101/255) of cases. Pre‐existing hypertension and diabetes were present in 3.9% (10/255) and 2.4% (6/255) of cases, respectively. The birthweight centile median (IQR) was 16.1 (0.89–53.5), and the gestational age median (IQR) at delivery was 38.0 (35.9–39.4) weeks. Pre‐eclampsia was diagnosed in 153 out of 255 (60%) HDP cases. There were eight cases of HELLP syndrome. Composite adverse maternal outcomes occurred in 26.7% (68/255). Table 1 shows the frequency of each adverse maternal outcome included. Median (IQR) differences in days between TTE and delivery were 6^3^, ^4^, ^5^, ^6^, ^7^, ^8^, ^9^, ^10^, ^11^, ^12^, ^13^, ^14^ days before delivery and 2^1^, ^2^, ^3^ days after delivery. 18.1% (46/255) of HDP patients underwent TTE within 1 week of delivery.

**TABLE 1:** Baseline maternal characteristics, antihypertensive treatments, and incidence of composite maternal outcomes in the cohort of pregnancies affected by hypertensive disorders of pregnancy (HDP).

Variables	Total HDP (*N* = 255)
Age (year)	33.8 (29.9–37.3)
1st trimester BMI (kg/m^2^)	27.0 (23.1–31.2)
1st trimester MAP	94.7 (90.3–99.3)
Ethnicity	
Caucasian	156 (60.4)
Afro‐Caribbean	48 (18.8)
Asian	40 (15.7)
Mixed/other	13 (5.1)
Smoker	27 (10.6)
IVF	16 (6.3)
Nulliparous	167 (65.5)
Pre‐existing CHTN	10 (3.9)
Pre‐existing DM	6 (2.4)
High risk of preterm pre‐eclampsia	70 (34.8)
Gestational age at delivery (weeks)	38.0 (35.9–39.4)
Birthweight centile	16.1 (0.89–53.5)
Perinatal death	4 (1.6)
Preterm birth <34 weeks	37 (14.5)
Antihypertensive treatments	
Labetalol	50 (19.6)
Nifedipine	77 (30.2)
Labetalol, nifedipine	50 (19.6)
Methyldopa	13 (5.1)
Labetalol, methyldopa	1 (0.4)
Nifedipine, methyldopa	11 (4.3)
Labetalol, nifedipine, methyldopa	5 (2.0)
Composite maternal outcome	68 (26.7)
HDU admission	54 (21.2)
Acute kidney injury	21 (8.2)
Stroke	1 (0.4)
Peripartum cardiomyopathy	1 (0.4)
Pulmonary oedema	1 (0.4)
Disseminated intravascular coagulation	1 (0.4)

*Note*: Values are presented as *n* (%) or median (interquartile range).

Abbreviations: BMI, body mass index; CHTN, chronic hypertension; DM, diabetes mellitus; HDP, hypertensive disorders of pregnancy; HDU, high‐dependency unit; MAP, mean arterial pressure; SGA, small for gestational age.

Women who had composite adverse outcomes delivered significantly earlier (gestational age 35.79 [31.6–38.3] weeks) and had smaller babies (birthweight centile 4.7 [0.1–39.3]) compared to those who did not (gestational age 38.6 [37.1–39.4] weeks, *p* < 0.001; birthweight centile 21.0 [2.3–60.7], *p* = 0.003). They also showed lower platelets and higher creatinine, ALT, and PCR compared to women who did not develop adverse maternal outcomes, as expected (Table 2). The univariate analysis, presented in Table 3, highlighted which echocardiographic parameters were significantly associated with adverse maternal outcomes in HDP pregnancies. Women with adverse maternal outcomes had higher LAVI (28.8 [23.4–32.3] mL/m^2^ vs. 26.6 [22.2–30.9] mL/m^2^, *p* = 0.045) and E/e' ratio (7.80 [6.59–9.23] vs. 7.00 [5.91–8.08], *p* = 0.002) compared to those without adverse outcomes. RV function showed that TAPSE was significantly higher in HDP women with adverse maternal outcomes (3.00 [2.65–3.30] vs. 2.80 [2.50–3.10], *p* = 0.018), whereas RV FAC did not change between groups (Table 2). These associations remained significant also after adjusting for maternal factors before TTE (maternal age, non‐white ethnicity, 1st trimester BMI, 1st trimester MAP, and pre‐existing comorbidities) and at the time of TTE (BMI, MAP, and timing of TTE, including gestational age or days postpartum if TTE was done after delivery), as shown in Table 3. In the sensitivity analysis, excluding women who underwent TTE after delivery, the associations between echocardiographic parameters and adverse maternal outcomes remained consistent with the main analysis (Supplementary Table 2). While 44 patients (17.3%) experienced only one adverse outcome, 29 patients (11.4%) had two or more adverse maternal outcomes. In the ordinal regression analysis, the E/e' ratio remained significant (OR 1.28, 95% CI 1.10–1.49, *p* = 0.001), while LAVI was not (OR 1.03, 95% CI 0.98–1.07, *p* = 0.250).

**TABLE 2:** Maternal and laboratory characteristics by adverse maternal outcome status in the HDP cohort.

Variables	Adverse maternal outcomes (*N* = 68)	No adverse maternal outcomes (*N* = 187)	|SMD|
Maternal characteristics			
Age (years)	33.2 (28.8–36.7)	34.1 (30.3–37.5)	0.107
1st trimester BMI (kg/m^2^)	26.7 (23.0–30.5)	27.2 (23.4–31.5)	0.173
1st trimester MAP (mmHg)	93.0 (89.0–99.3)	95.3 (90.7–99.3)	0.014
Non‐white Ethnicity	32 (47.1)	69 (36.9)	**0.208**
1st trimester high‐risk preterm pre‐eclampsia	17 (37.8)	53 (34.0)	0.080
Pre‐existing CHTN	2 (2.9)	8 (4.3)	0.069
Pre‐existing DM	2 (2.9)	4 (2.1)	0.053
Gestational age at diagnosis	34.0 (30.0–37.0)	36.4 (34.0–38.0)	**0.230**
BMI (kg/m^2^) at TTE	30.8 (26.6–33.6)	31.3 (27.9–35.6)	**0.237**
MAP (mmHg) at TTE	104.7 (99.7–108.8)	103.7 (98.7–108.7)	**0.224**
Laboratory parameters			
Platelet count (10^9^/L)	179.5 (130.0–214.0)	210.0 (172.0–250.0)	**0.735**
Creatinine (μmol/L)	71.0 (62.0–87.0)	61.0 (52.0–71.0)	**0.812**
ALT (U/L)	26.0 (17.0–62.0)	19.0 (14.0–28.0)	**0.655**
PCR (mg/mmol)	133.8 (55.0–459.8)	37.2 (13.6–94.9)	**1.007**

*Note*: Values are median [IQR] or *n* (%); no hypothesis testing performed. SMD, standardized mean difference; imbalance categories by |SMD|: none <0.10, minimal 0.10–0.19, relevant ≥0.20. Bold values indicate relevant SMD >0.20.

Abbreviations: ALT, alanine aminotransferase; BMI, body mass index; CHTN, chronic hypertension; DM, diabetes mellitus; HDP, hypertensive disorders of pregnancy; MAP, mean arterial pressure; PCR, protein:creatinine ratio; TTE, transthoracic echocardiography.

**TABLE 3:** The association between echocardiographic parameters and adverse maternal outcomes, adjusted for maternal demographic and clinical characteristics.

Variables	Adverse maternal outcomes (*N* = 68)	No adverse maternal outcomes (*N* = 187)	*p* value	OR (95% CI)	*p* value	OR^a^ (95% CI)	*p* value	OR^b^ (95% CI)	*p* value
LVMI (g/m^2^)	78.3 (69.0–92.2)	77.03 (66.3–86.2)	0.167	1.02 (1.00–1.04)	0.053	—	—	—	—
RWT	0.43 (0.36–0.47)	0.43 (0.36–0.48)	0.528	2.56 (0.13–51.3)	0.540	—	—	—	—
LAVI (mL/m^2^)	28.8 (23.4–32.3)	26.6 (22.2–30.9)	**0.045**	1.05 (1.01–1.10)	**0.024**	1.07 (1.02–1.12)	**0.010**	1.06 (1.01–1.11)	**0.025**
Lateral e' (m/s)	0.13 (0.10–0.14)	0.12 (0.11–0.15)	0.330	0.94 (0.85–1.03)	0.208	—	—	—	—
Septal e' (m/s)	0.09 (0.08–0.12)	0.10 (0.08–0.12)	0.364	0.96 (0.85–1.07)	0.431	—	—	—	—
E/A	1.25 (0.95–1.41)	1.23 (1.07–1.38)	0.973	0.91 (0.34–2.39)	0.843	—	—	—	—
E/e'	7.80 (6.59–9.23)	7.00 (5.91–8.08)	**0.002**	1.28 (1.10–1.49)	**0.002**	1.29 (1.10–1.52)	**0.002**	1.30 (1.10–1.52)	**0.002**
EF (%)	59.0 (57.00–61.0)	58.0 (55.0–61.0)	0.152	1.04 (0.97–1.11)	0.285	—	—	—	—
GLS (%)	−16.3 (−17.8‐‐14.5)	−16.15 (−17.9‐‐14.5)	0.852	0.98 (0.88–1.09)	0.670	—	—	—	—
FAC	45.62 (39.74–53.85)	47.01 (40.74–52.23)	0.780	0.99 (0.96–1.02)	0.577	—	—	—	—
S′	0.16 (0.15–0.19)	0.15 (0.14–0.18)	**0.048**	1.1 (1.0–1.21)	**0.044**	1.1 (1.0–1.22)	0.056	1.11 (1.01–1.22)	**0.030**
TAPSE	3.00 (2.65–3.30)	2.80 (2.50–3.10)	**0.018**	2.31 (1.22–4.36)	**0.010**	2.49 (1.26–4.93)	**0.009**	2.24 (1.18–4.27)	**0.014**

*Note*: Results include odds ratios (OR) with 95% confidence intervals (CI). Values are median [IQR]. Bold values indicate relevant SMD >0.20.

Abbreviations: 95% CI, confidence interval; EF, ejection fraction; FAC, fractional area change; GLS, global longitudinal strain; LAVI, left atrial volume index; LVMI, left ventricular mass index; OR, odd ratio; RWT, relative wall thickness; TAPSE, tricuspid annular plane systolic excursion.

^a^ Adjusted for maternal age, non‐white ethnicity, 1st trimester BMI, 1st trimester MAP, and pre‐existing comorbidities.

^b^ Adjusted for BMI, MAP, and timing of echocardiography (gestational age, before or after delivery).

## 4 DISCUSSION

Pregnancies complicated by HDP and adverse maternal outcomes demonstrated significantly higher LAVI and E/e' values, underscoring the association between diastolic dysfunction and maternal morbidity in this high‐risk population. In particular, E/e' was also associated with a more severe phenotype of adverse maternal outcomes. Women with adverse maternal outcomes also showed higher TAPSE and S' but unchanged FAC, consistent with a hyperkinetic circulatory state rather than improved RV function. These findings indicate that HDP patients who develop adverse maternal outcomes could benefit from closer cardiac monitoring during the peripartum and optimal postnatal management to improve their cardiovascular outcomes.^23^

The findings we observed are physiologically plausible, as left ventricular remodeling of cardiac structure and impaired diastolic function are common features in pregnancies affected by HDP.^9^, ^10^ There is a clear dose–response relationship between the severity of HDP and echocardiographic abnormalities, reinforcing this study's results.^11^ Alhuneafat and colleagues demonstrated that women with chronic hypertension and superimposed pre‐eclampsia exhibited the most severe abnormalities, including significantly elevated LVMI and E/e' ratio; women with pre‐eclampsia showed intermediate changes, and those with gestational hypertension exhibited the mildest changes, often not significantly different from normotensive controls after adjustment for confounders.^11^ However, few prior studies have linked cardiac dysfunction detected during pregnancies complicated by HDP with adverse maternal outcomes. In a prospective observational study, Vaught et al. found that women with pre‐eclampsia with severe features demonstrated higher right ventricular systolic pressures (31.0 ± 7.9 mmHg vs. 22.5 ± 6.1 mm Hg, *p* < 0.001), abnormal left ventricular diastolic parameters (septal E/e' ratio: 10.8 ± 2.8 vs. 7.4 ± 1.6, left atrial area size: 20.1 ± 3.8 cm^2^ vs. 17.3 ± 2.9 cm^2^), and increased left‐sided cardiac remodeling compared to healthy pregnant controls. Notably, within the pre‐eclamptic group, six women (9.5%) developed peripartum pulmonary oedema, further emphasizing the association between cardiac dysfunction and adverse outcomes.^12^

Previous work has reported apparently conflicting results regarding right ventricular systolic function in hypertensive disorders of pregnancy.^11^, ^12^ In our cohort, HDP women with adverse outcomes showed higher TAPSE but unchanged FAC, indicating that the greater longitudinal excursion of the tricuspid annulus does not translate into improved global RV systolic performance. These findings are more consistent with a load‐related hyperkinetic state rather than enhanced intrinsic contractility. Vaught et al. demonstrated that women with severe pre‐eclampsia exhibited increased TAPSE but reduced RV longitudinal strain on speckle‐tracking, revealing subclinical RV dysfunction that is not apparent from conventional indices.^12^ As our study did not include strain analysis, further research incorporating advanced imaging is needed to clarify the true impact of HDP on RV myocardial mechanics.

Our study demonstrated a more significant cardiovascular impact, particularly on diastolic function, in HDP pregnancies with maternal adverse outcomes compared to those without such complications. This highlights a potential need for close cardiac monitoring, including TTE, in this subgroup of HDP patients during the peripartum period, as they may be more likely to require cardiac support. Whether these changes are present prior to the onset of adverse maternal outcomes is also of interest as, if so, echocardiographic findings in HDP might assist in the early identification of women at high risk of developing maternal complications^24^, ^25^, ^26^ At present, risk in hypertensive pregnancies may be assessed using the full Ρrееclampsia Integrated Estimate of Risk model. This is a validated predictive tool designed to estimate the risk of adverse maternal outcomes in women with pre‐eclampsia, including eclampsia, stroke, pulmonary oedema, renal failure, liver dysfunction, disseminated intravascular coagulation, placental abruption, and maternal death within 48 h or 7 days.^27^ It integrates clinical parameters, such as gestational age and symptoms (chest pain and dyspnea), oxygen saturation, and laboratory parameters, including liver function, creatinine, and platelet count. After external validation, it demonstrated moderate predictive accuracy, with a pooled AUC of 0.78 (95% CI 0.71–0.86) for outcomes within 48 h and 0.75 (95% CI 0.69–0.82) within 7 days, with better performance in high‐income countries than low‐ and middle‐income countries.^24^ Whether the inclusion of echocardiographic parameters in a predictive tool could improve the detection of pre‐eclamptic women at risk of adverse maternal outcomes requires further work.

Evidence shows that women with HDP who exhibit worse cardiac remodeling and diastolic dysfunction are at a significantly higher risk of developing chronic hypertension as early as 4–6 months postpartum.^13^ Chronic hypertension, in turn, is the primary risk factor for subsequent cardiovascular complications, such as heart failure and myocardial infarction, in the female population.^28^, ^29^ The Physician Optimized Postpartum Hypertension Treatment (POP‐HT) trial has provided compelling evidence of the importance of structured interventions during the postpartum period. In this trial, 220 women with HDP were randomized to either physician‐guided self‐monitoring of blood pressure with optimized antihypertensive medication titration or standard postpartum care. The intervention group showed significantly better blood pressure control at 6–9 months postpartum.^23^ Moreover, echocardiographic and cardiac magnetic resonance imaging revealed that the intervention group experienced improved cardiac remodeling, including reductions in left ventricular mass, end‐diastolic and end‐systolic volumes, and relative wall thickness, alongside improvements in left and right ventricular ejection fractions.^30^ These findings underscore the critical role of early and intensive postpartum management in improving outcomes for women with HDP. Whether the more extreme cardiac changes we observed in those who had adverse maternal outcomes in the current study persist postpartum and can be reversed by improved postpartum care will be of interest.

The strengths of this study include its prospective design, rigorous echocardiographic assessments, and a well‐defined cohort of women with HDP with ethnic diversity. TTE were acquired under a research protocol and kept blinded to clinicians, minimizing treatment bias. However, there are several limitations. The study was conducted at a single tertiary center, potentially limiting generalizability to other populations. Furthermore, although this study is a subanalysis of a cohort initially designed to assess postpartum outcomes, the study is not adequately powered to evaluate adverse maternal outcomes as a primary predictor of future endpoints, and our analysis was limited to a case–control analysis.^13^, ^14^, ^15^ The small sample size, particularly for severe adverse events, also restricted the statistical power to detect additional significant associations or associations with specific individual adverse outcomes. Lastly, the lack of standardized management protocols across participants may have introduced variability in clinical outcomes and intervention bias. Only a small proportion of women (*n* = 10) had pre‐existing chronic hypertension, which could have influenced echocardiographic parameters such as LAVI and E/e', although the small numbers precluded meaningful subgroup analyses. Moreover, we could not ascertain whether every peripartum TTE preceded the adverse outcome, so estimates reflect associations rather than causation. Despite these limitations, the findings underscore the potential utility of integrating TTE into clinical practice for managing HDP pregnancies in the peripartum and postpartum period.

## 5 CONCLUSION

This study highlights a significant potential role for echocardiographic parameters, particularly LAVI and E/e' ratio, to identify women at risk of adverse maternal outcomes in pregnancies complicated by HDP. These findings advocate for further prospective work to understand whether the integration of cardiac assessments into routine peripartum clinical management for HDP at risk of adverse maternal outcomes could allow for targeted monitoring and timely interventions to mitigate peripartum adverse maternal outcomes. Echocardiographic findings obtained during the peripartum could also inform targeted postnatal interventions to improve cardiovascular health and reduce long‐term risk.

## AUTHOR CONTRIBUTIONS

VG conducted the study for her PhD, analyzed the data, and wrote the first draft of the article; JK, AK, PL, JOD, and BT critically reviewed the article.

## FUNDING INFORMATION

Veronica Giorgione received funding from the European Union's Horizon 2020 research and innovation programme under the Marie Skłodowska‐Curie Grant Agreement No. 765274 (iPLACENTA project).

## CONFLICT OF INTEREST STATEMENT

The authors report no conflict of interest.

## ETHICS STATEMENT

Ethical approval was obtained on the February 01, 2019 from the Local Ethics Committee (19/LO/0794), and all participants provided written informed consent.

## Supporting information


**Table S1.** Relative risks (RR) of adverse maternal outcomes associated with peripartum TTE parameters (unadjusted, Model A, Model B).
**Table S2.** Comparison of echocardiographic parameters in HDP women with and without adverse maternal outcome before delivery (*n* = 209).
**Table S3.** Adjusted odds ratios (OR) with 95% CI for the association between TTE parameters and adverse maternal outcomes.

## Data Availability

The data that support the findings of this study are available from the corresponding author upon reasonable request.
