# Supplementary material for: Cardiac dysfunction during adverse maternal outcomes in hypertensive disorders of pregnancy
Source: Acta Obstet Gynecol Scand. 2025 Nov 17;105(2):280–7. doi: 10.1111/aogs.70103 (PMC12856716; doi:10.1111/aogs.70103)
Supplement: Supplementary file 1 — Table S1. Relative risks (RR) of adverse maternal outcomes associated with peripartum TTE parameters (unadjusted, Model A, Model B). Table S2. Comparison of echocardiographic parameters in HDP women with and without adverse maternal outcome before delivery (n = 209). Table S3. Adjusted odds ratios (OR) with 95% CI for the association between TTE parameters and adverse maternal outcomes. [file AOGS-105-280-s001.docx]

**Supplementary Tables**

**Table S1. Relative risks (RR) of adverse maternal outcomes associated with peripartum TTE parameters (unadjusted, Model A, Model B)**

| **Variable** | **RR** | **p value** | **RR^a^ (95% CI)** | **p value** | **RR^b^ (95% CI)** | **p value** |
| --- | --- | --- | --- | --- | --- | --- |
| LVMI (g/m²) | 1.01 (1.00–1.02) | 0.043 | 1.01 (1.00–1.03) | 0.073 | 1.01 (1.00–1.02) | 0.049 |
| RWT | 1.97 (0.24–16.26) | 0.528 | 1.44 (0.14–14.48) | 0.758 | 2.25 (0.27–18.61) | 0.451 |
| LAVI (ml/m²) | 1.04 (1.01–1.07) | 0.021 | 1.05 (1.01–1.08) | 0.012 | 1.04 (1.01–1.07) | 0.023 |
| MV E/A | 0.93 (0.44–1.98) | 0.851 | 0.78 (0.34–1.80) | 0.566 | 0.89 (0.42–1.87) | 0.758 |
| Lateral e′ (m/s) | 0.96 (0.90–1.02) | 0.175 | 0.96 (0.89–1.03) | 0.279 | 0.95 (0.89–1.01) | 0.103 |
| Septal e′ (m/s) | 0.97 (0.89–1.05) | 0.443 | 0.97 (0.88–1.06) | 0.510 | 0.96 (0.88–1.05) | 0.361 |
| E/e′ | 1.17 (1.08–1.25) | <0.001 | 1.17 (1.08–1.27) | <0.001 | 1.17 (1.09–1.26) | <0.001 |
| EF (%) | 1.03 (0.98–1.08) | 0.277 | 1.02 (0.97–1.08) | 0.433 | 1.02 (0.97–1.07) | 0.474 |
| GLS (%) | 0.98 (0.91–1.06) | 0.663 | 0.98 (0.90–1.07) | 0.694 | 0.99 (0.92–1.07) | 0.868 |
| TAPSE (cm) | 1.79 (1.21–2.65) | 0.003 | 1.84 (1.26–2.69) | 0.002 | 1.72 (1.19–2.48) | 0.004 |
| RV s′ (m/s) | 1.01 (1.01–1.01) | <0.001 | 1.01 (1.01–1.01) | <0.001 | 1.01 (1.01–1.01) | <0.001 |
| RV FAC (%) | 0.99 (0.97–1.02) | 0.588 | 0.99 (0.97–1.02) | 0.623 | 0.99 (0.97–1.02) | 0.531 |

LVMI left ventricular mass index, RWT relative wall thickness, LAVI left atrial volume index, EF ejection fraction, GLS global longitudinal strain, TAPSE Tricuspid Annular Plane Systolic Excursion, FAC Fractional Area Change.

*Model^a^: adjusted for age, non-white ethnicity, booking BMI, booking MAP, pre-diabetes, and chronic hypertension.*

*Model^b^: adjusted for BMI, MAP at TTE, and TTE timing.*

**Table S2. Comparison of echocardiographic parameters in HDP women with and without adverse maternal outcome before delivery (n=209).**

| **TTE parameter** | **Adverse maternal outcomes**  **(n=53)** | **No adverse maternal outcomes**  **(n=156)** | **P value** | **OR (95% CI)** |
| --- | --- | --- | --- | --- |
| LVMI (g/m2) | 77.15 (68.99–90.83) | 76.99 (64.82–85.71) | 0.258 | 1.02 (1.00–1.04) |
| RWT | 0.43 (0.37–0.48) | 0.42 (0.35–0.48) | 0.306 | 1.16 (0.84–1.59) |
| LAVI (ml/m2) | 28.94 (23.59–32.68) | 26.34 (22.19–29.77) | **0.014** | 1.08 (1.02–1.15) |
| Lateral e’ (m/s) | 0.12 (0.10–0.14) | 0.12 (0.11–0.14) | 0.366 | 0.94 (0.84–1.04) |
| Septal e’ (m/s) | 0.09 (0.08–0.11) | 0.10 (0.08–0.12) | 0.094 | 0.90 (0.79–1.03) |
| E/e′ | 8.00 (6.73–9.43) | 6.90 (5.91–8.28) | **0.002** | 1.30 (1.10–1.54) |
| EF (%) | 59.00 (57.00–61.00) | 58.00 (56.00–61.00) | 0.525 | 1.02 (0.95–1.11) |
| GLS (%) | -16.17 (-17.80–-14.45) | -16.20 (-18.10–-14.50) | 0.875 | 0.98 (0.87–1.11) |

LVMI left ventricular mass index, RWT relative wall thickness, EF ejection fraction, GLS global longitudinal strain, FAC Fractional Area Change.

**Table S3.** **Adjusted odds ratios (OR) with 95% CI for the association between TTE parameters and adverse maternal outcomes**

| **Variables** | **OR^a^ (95% CI)** | **P value** | **OR^b^ (95% CI)** | **P value** |
| --- | --- | --- | --- | --- |
| LVMI (g/m²) | 1.02 (1.00–1.04) | 0.086 | 1.02 (1.00–1.04) | 0.062 |
| RWT | 1.65 (0.06–48.10) | 0.772 | 3.10 (0.14–68.24) | 0.474 |
| MV E/A | 0.72 (0.24–2.14) | 0.551 | 0.87 (0.32–2.36) | 0.778 |
| Lateral e′ (m/s) | 0.95 (0.85–1.05) | 0.313 | 0.93 (0.84–1.03) | 0.145 |
| Septal e′ (m/s) | 0.96 (0.85–1.09) | 0.508 | 0.95 (0.84–1.07) | 0.379 |
| EF (%) | 1.03 (0.96–1.11) | 0.437 | 1.03 (0.96–1.10) | 0.466 |
| GLS (%) | 0.98 (0.87–1.10) | 0.693 | 0.99 (0.88–1.11) | 0.856 |
| RV FAC (%) | 0.99 (0.96–1.03) | 0.620 | 0.99 (0.96–1.02) | 0.517 |

LVMI left ventricular mass index, RWT relative wall thickness, EF ejection fraction, GLS global longitudinal strain, FAC Fractional Area Change.

*Model^a^: adjusted for age, non-white ethnicity, booking BMI, booking MAP, pre-diabetes, and chronic hypertension.*

*Model^b^: adjusted for BMI, MAP at TTE, and TTE timing.*
